# Thirty-eight-year follow-up of the first patient of mandibular reconstruction with free vascularized fibula flap

**DOI:** 10.1186/s13005-021-00293-z

**Published:** 2021-10-28

**Authors:** Edela Puricelli, Roberto Correa Chem

**Affiliations:** 1 Oral and Maxillofacial Surgery Unit, School of Dentistry, Clinical Hospital of Porto Alegre (HCPA), Federal University of Rio Grande do Sul (UFRGS), Rua Ramiro Barcelos, RS 2492-90035-003 Porto Alegre, Brazil; 2 Department of Plastic Surgery and Reconstructive Microsurgery, Santa Casa de Misericordia de Porto Alegre, Rua Professor Annes Dias , RS 295-90020-090 Porto Alegre, Brazil

**Keywords:** Mandibular reconstruction, Free vascularized fibula graft, Microsurgery, Long-term follow-up

## Abstract

**Background:**

The mandible is responsible for vital functions of the stomatognathic system, and its loss results in functional and aesthetic impairment. Mandibular reconstruction with free fibula flap is considered the gold standard for mandibular reconstruction.

**Case presentation:**

We describe here the 38-year follow-up of the patient who was the first case of mandibular reconstruction with free fibula flap reported in the literature. The original report describes a 27-year-old woman who had undergone extensive mandibulectomy due to an osteosarcoma. A microvascularized fibula flap was used for mandibular reconstruction in 1983. Two years later, a vestibulo-lingual sulcoplasty with skin graft was performed to allow the construction of a total dental prosthesis. Fifteen years after the initial treatment, an autologous iliac crest graft was placed in the fibula flap, aimed at increasing bone thickness and height for rehabilitation with implant supported prosthesis. In 2015, a rib graft was positioned in the mental region, enhancing the support to the soft tissues of the face and improving the oral function. A recent review of the patient shows well-balanced facial morphology and optimal functional results of the procedure.

**Conclusions:**

The fibula flap method, described in 1975 and first reported for mandibular reconstruction in 1985, continues to be applied as originally described, especially where soft tissue damage is not extensive. Its use in reconstructive surgery was expanded by advancements in surgery and techniques such as virtual surgical planning. However, there is still a lack of evidence related to the long-term evaluation of outcomes. The present work represents the longest-term follow-up of a patient undergoing mandibular reconstruction with free vascularized fibula flap, presenting results showing that, even after 38 years, the procedure continues to provide excellent results.

## Background

The mandible participates in vital functions of the stomatognathic system, such as mastication, respiration, swallowing, and phonation. Mandibular loss, which may be due to trauma, infections such as osteomyelitis, or resection of benign or malignant tumors, results in functional and aesthetic impairment, directly interfering in the patient’s quality of life. Full reconstruction of the mandible, therefore, is necessary for the recovery of both aesthetics and function [1].

Reconstruction of mandibular defects remain a major challenge to oral and maxillofacial surgeons. Approaches including the use of alloplastic biocompatible plates and meshes [2, 3], as well as distraction osteogenesis [4], have already been described, however, intra- and postoperative complications have been reported [5].

Although attempts at mandibular reconstruction using bone grafts have long been described, the first great advances were made during the First and Second World Wars [6]. Initially, free non-vascularized bone grafts were employed, however, the emergence of vascular surgery allowed the use of pedicled flaps that kept their connection with the donor site [7]. Finally, improvements in microsurgical techniques and technological advancements led to the replacement of these two approaches with free vascularized bone flaps for mandibular reconstruction.

In 1975, Taylor et al. first reported the use of a free fibula flap in the treatment of a post-traumatic tibial defect [8]. The posterior harvesting technique initially used was further improved by description of a lateral approach [9], allowing direct visualization of the peroneal artery and safe inclusion of a skin paddle with the bone flap. In 1989, Hidalgo described mandibular reconstruction with free fibula flap in 12 patients [10]. Since then, the fibula free flap has been considered the gold standard for mandibular reconstruction; due to its length of around 20 to 25 cm, it can be used as an osseous, osteocutaneous, or osteoseptocutaneous free flap and donor-site morbidity is not significant.

We herein present the 38-year follow-up of the patient who was the first case of mandibular reconstruction with free fibula flap reported in the literature [11]. This longest-term follow-up of a patient undergoing this treatment significantly adds to our knowledge of the use of free vascularized fibula flap (FVFF) in mandibular reconstruction.

### Case presentation

**Fig. 1:**
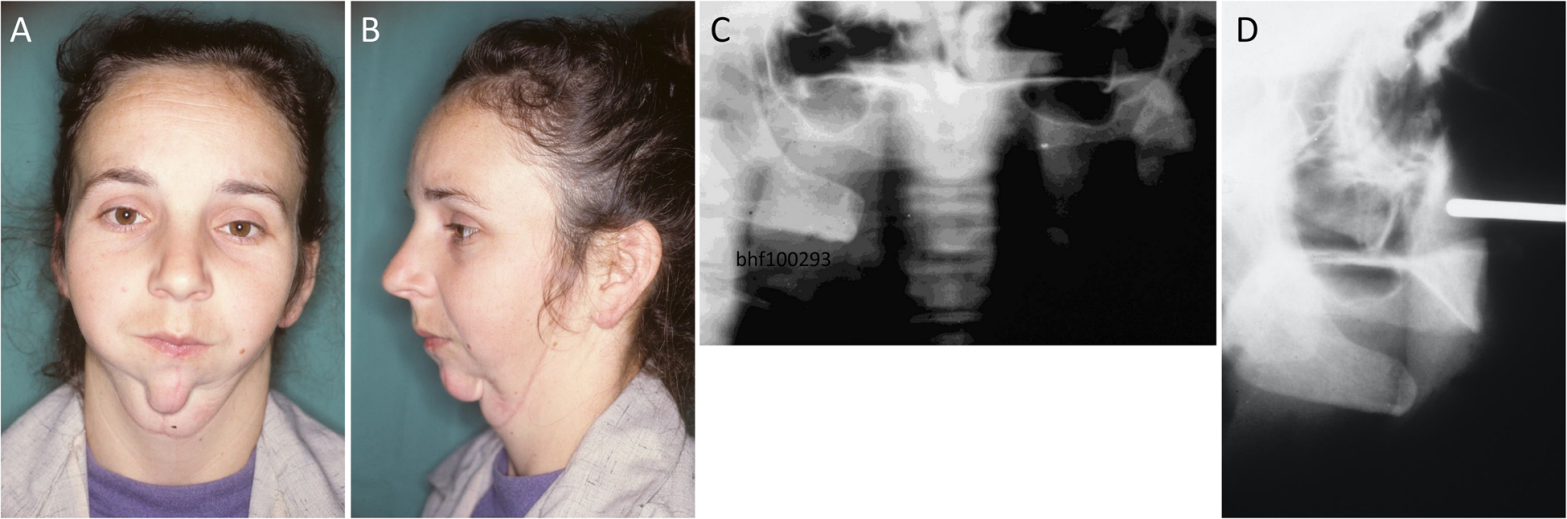
Preoperative images. **a, b** Frontal and lateral facial aspects of the patient, showing extensive deformity in the lower third of the face as a result of mandibulectomy. **c** A panoramic radiograph shows, on the right side, the ramus, the angle of the mandible and the retromandibular area; on the left side, remnants of the ascending ramus, from the mandibular foramen on. (Reproduced from Puricelli and Chem, 1985 [11], with permission from RGO – Revista Gaucha de Odontologia, ISSN 0103–6971). **d** Facial teleradiography

The original case was described by Puricelli and Chem in 1985 [11]. Briefly, a 27-year-old woman was referred to us for facial reconstruction. Five years earlier, she had undergone extensive mandibulectomy due to an osteosarcoma (Fig. 1a,b). Clinical examination revealed a submandibular incision extending from the right to the left angle of the mandible, as well as a second vertical incision extending from the lower lip to the level of the hyoid bone. She was completely edentulous. Radiographic examinations showed absence of the mandibular body and remnants of the ascending ramus; the temporomandibular joints were functional.

**Fig. 2:**
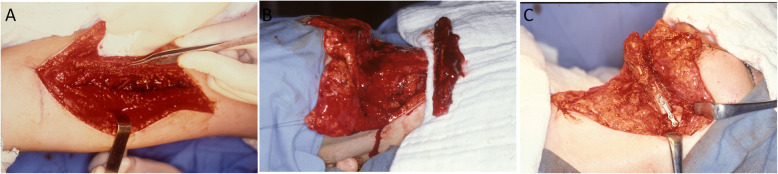
Transoperative images. **a** Harvesting of the FVFF on the right leg. **b** Preparation of the receptor area; the straight fibula flap, with soft tissues including the vascular pedicle, can be seen. **c** V-shaping of the FVFF and fixation with titanium 2.0 miniplates (left side view)

For mandibular reconstruction, a microvascularized fibula flap was used. The surgical procedure was initiated by a submandibular incision similar to the prior incision, in order to achieve similar wide exposure of the surgical field and the right and left bone segments. Simultaneously, a 16-cm free fibula flap, accompanied by the peroneal artery and two veins, was harvested and modeled into a V shape while maintaining the attached soft tissue (Fig. 2a,b). After preparation of the receptor sites, the FVFF was fixed in place with a 2.0 mm Champy´s titanium miniplate, with space and four holes on the left side and six holes on the right side (Fig. 2c).

**Fig. 3:**
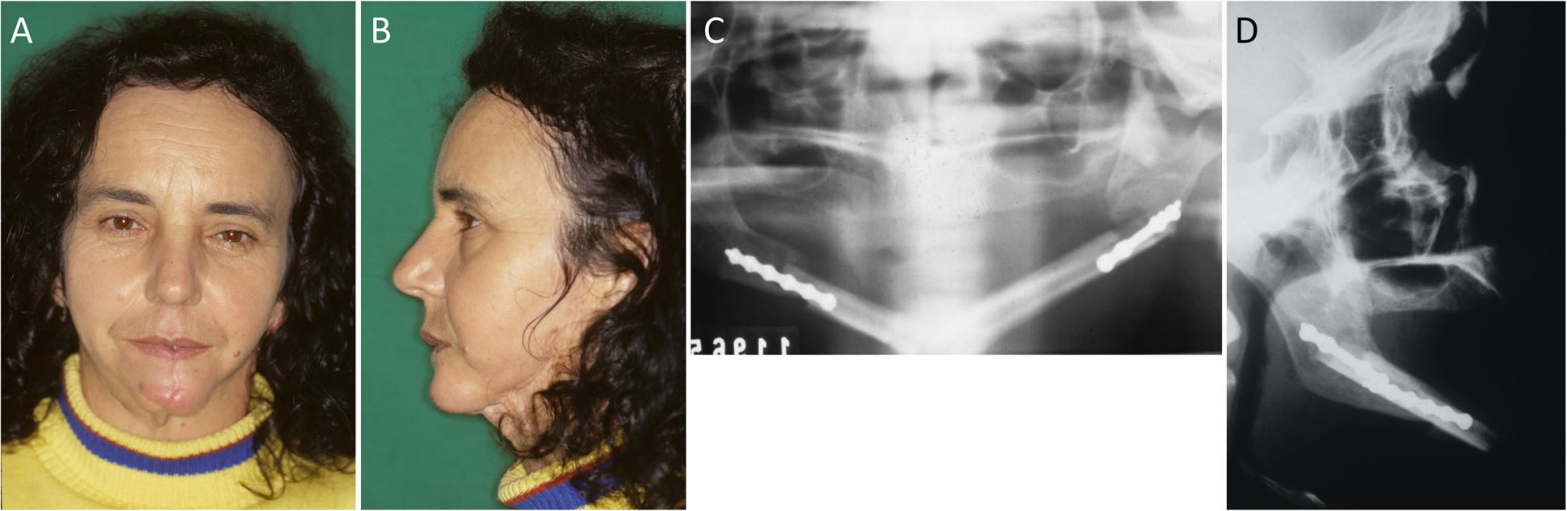
Postoperative images. **a, b** Frontal and lateral facial postoperative aspects. **c, d** Postoperative panoramic and facial teleradiographs with the fibula bone graft

The peroneal artery was anastomosed to the facial artery while the peroneal vein was anastomosed to the external jugular vein, both on the right side. When adequate blood supply of the flap was confirmed, the surgical field was closed by layers and a compressive dressing was applied. Postoperative care included the use of medication for pain, infection, edema, and thrombosis. The mandible was allowed to function 48 h postoperatively (Fig. 3).

**Fig. 4:**
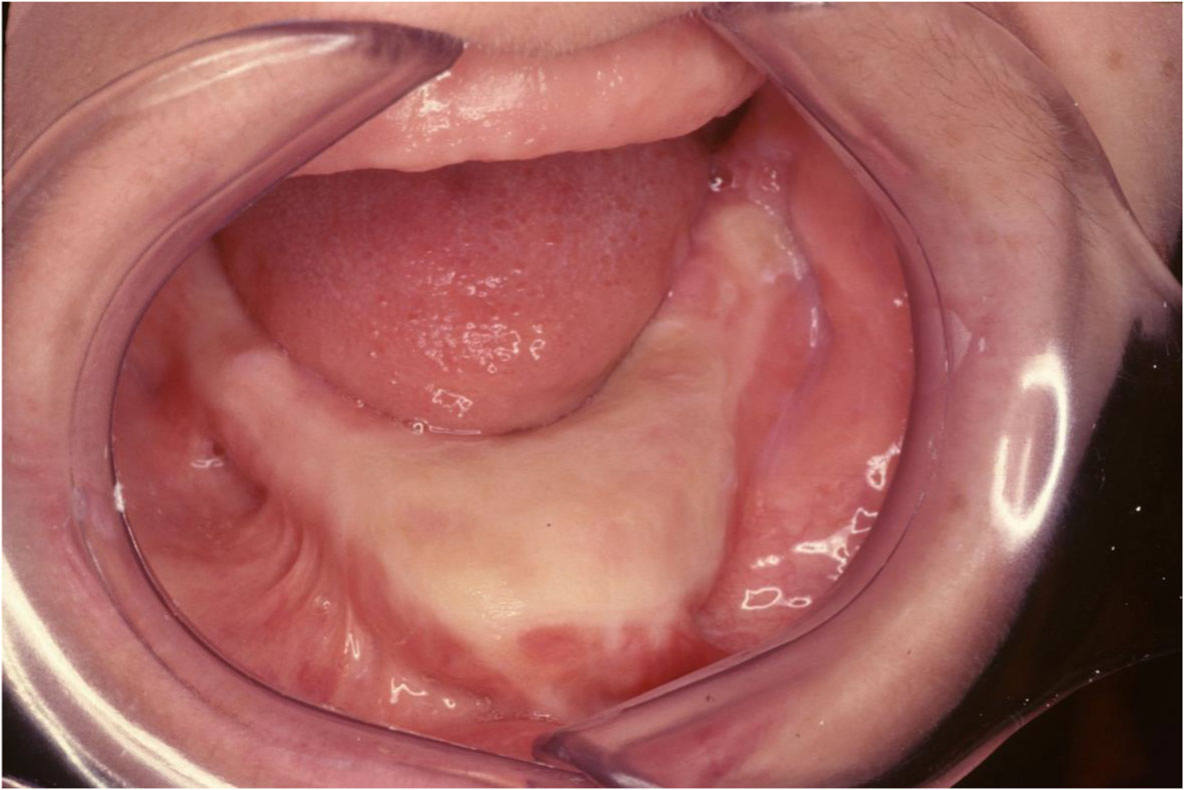
Intraoral view. Alveolar crest reconstructed after a vestibulo-lingual sulcoplasty with skin graft

Two years later, a vestibulo-lingual sulcoplasty with skin graft was performed in order to modify the soft tissue insertion sites and increase the stability of a total dental prosthesis (Fig. 4). The two rigid internal fixations placed in the first surgery were removed during this intervention.

**Fig. 5:**
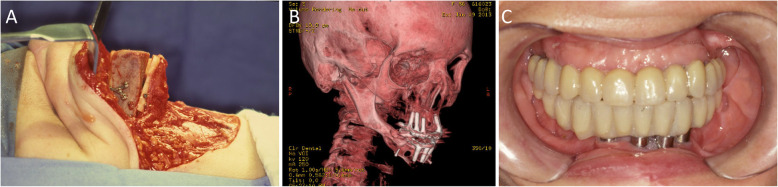
Grafting of the iliac crest bone flap in the fibula flap. **a** Transoperative image. The autologous iliac crest graft, which represented around 3–4 times the original fibula flap, was fixed with Champy’s mini/microplates within the fibular structure at the anterior mandibular region using the sandwich technique. **b** Facial 3D computed tomography (CT), revealing the osseointegration of the dental implants. **c** Placement of implant-supported protocol-type prosthesis

In 1998, 15 years after the initial treatment, advancements in preprosthetic surgery and osseointegrated implants allowed placement of an iliac crest graft in the fibula flap at the anterior mandibular region, aimed at increasing bone thickness and height for rehabilitation with an implant supported prosthesis (Fig. 5a-c).

**Fig. 6:**
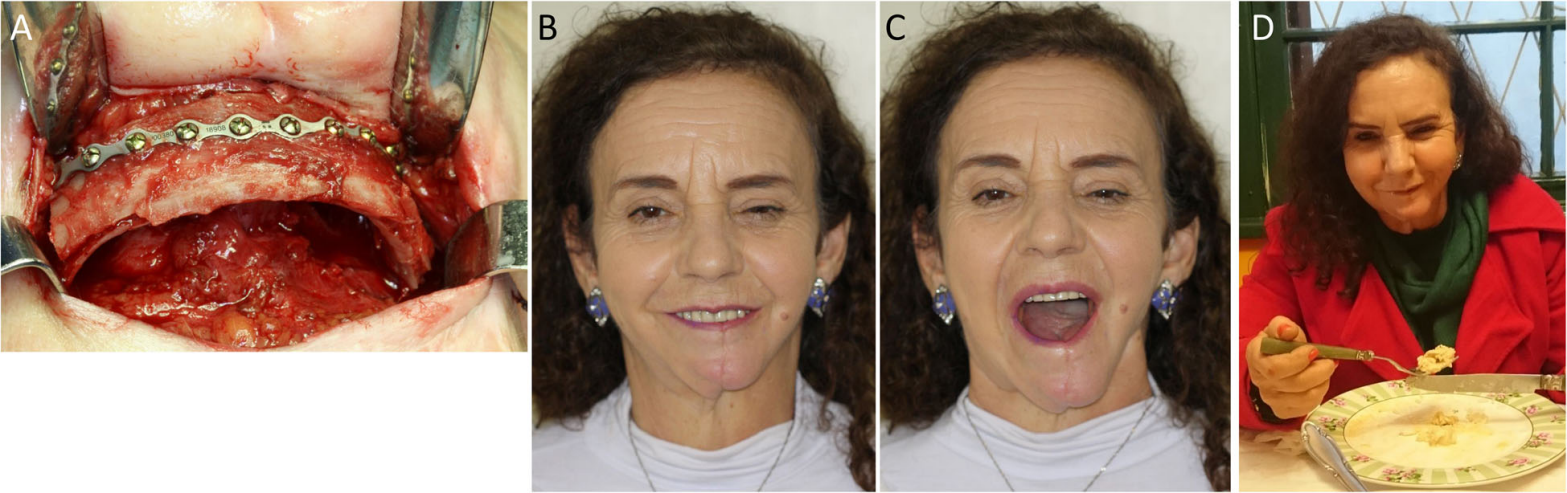
Postoperative aspects, in long-term follow of mandibular reconstruction with FVFF. **a** Transoperative image, placement of a rib flap in the mental region. **b, c** Facial aspects, improvement of mouth opening and oral motricity. **d** Social reinsertion through preservation of masticatory function

In 2015, an autologous rib graft was positioned in the mental region, enhancing the support to the soft tissues of the face and improving oral function (Fig. 6a). The perioral region has great functional and aesthetic importance, and its reconstruction may be complex; deformities may be apparent, either at rest or in movement, due to the soft and symmetrical nature of the tissue. The rib graft combines a cortical region, which gives support to soft tissues, and a medullary region, as a source of osteocompetent cells. The results showed preservation of smile, mouth opening without lateral deviation, and social reintegration of the patient, along with control of salivation, speech, and mastication (Fig. 6b-d).

**Fig. 7:**
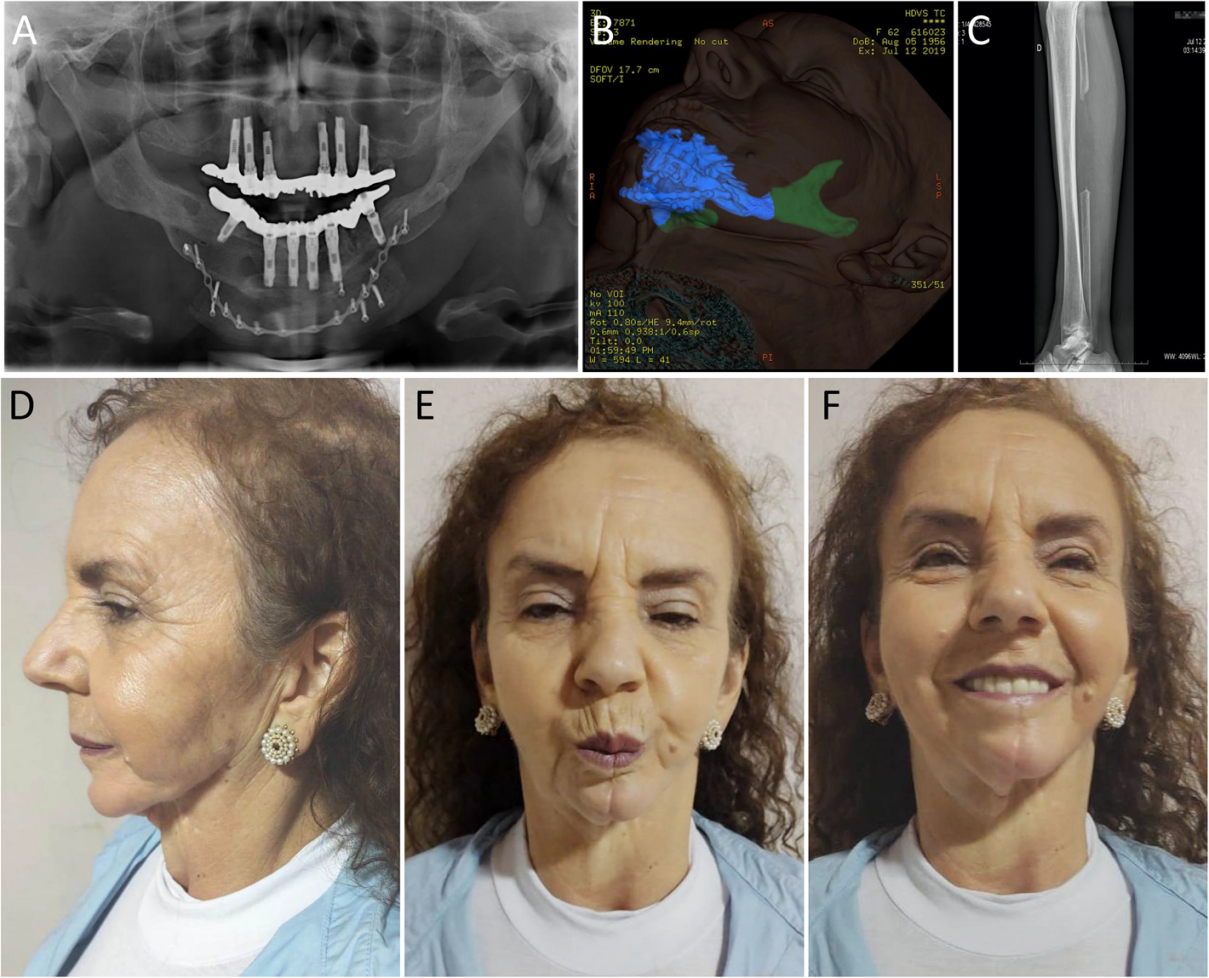
Postoperative aspects, in long-term follow-up of mandibular reconstruction with FVFF. **a** Panoramic radiograph showing implant-supported protocol-type prosthesis and a 16-hole 2.0 long plate used for fixation of the rib graft. **b** Facial 3D computed tomography (CT), showing the integration of the fibula, iliac crest and rib grafts (in blue) to the mandible remnant (in green). **c** Radiograph of the donor area, right leg. **d, e, f** Facial aspects. Images from 2021

Evaluations of the patient over the past few years have shown no morbidity of the donor site (Fig. 7a), accompanied by integration of the fibula, iliac crest, and rib grafts to the mandible remnants (Fig. 7b) and rehabilitation with an implant-supported protocol-type prosthesis (Fig. 7c). Thirty-eight years after performing mandibular reconstruction with FVFF, the patient was asked to describe her experience with regard to the functional results of the procedure. She presents a well-balanced facial morphology (Fig. 7d-f) and reports “very little” difficulty in the pronunciation of some words. No difficulty has been described in moving the head in any direction. She lives a healthy and high-quality life, which would have been impossible without the mandibular reconstruction performed long ago.

## Discussion and conclusions

The present case of mandibular reconstruction with FVFF was originally published in 1985 [11] and presented to the scientific community in 1986 and 1990 [12, 13]. At the time, fibula flaps had been used to treat lower limb injury [8], however, no report of mandibular reconstruction was available in the literature.

In the first proposal of use of fibula grafts, Taylor, Miller, and Ham [8] emphasized as their main positive points, in addition to a long and straight structure, the thick cortex and a viable vascular pedicle, which add to the low morbidity of the donor site. The main blood supply to the fibula is from the peroneal artery and multiple segmental musculoperiosteal vessels, while its venous drainage occurs through the two concomitant veins. The procedure involves two surgical teams working simultaneously and has a long duration, between 10 and 12 h. This first reconstructive surgery, reported herein, lasted for 14 h, which was considered adequate by the teams and produced favorable results, both in the immediate postoperative period and in medium- and long-term outcomes. The patient remained systemically stable in the immediate postoperative period, with no complications or complaints in the operated areas. The graft was buried in the receptor area and monitored for the first 72 h via evaluations based on skin color, temperature, and pulse rate in the surrounding area, as well as blood color and flow after local puncturing. In addition to routine postoperative medication, dextran IV was administered in the first 48 h [8].

In order to shape the graft to the recipient site, we confirmed similarity, in transverse sections, between the irregular oval profile of the fibular and mandibular bodies, with the exception of the dimensions. The posterior face of the graft mimics the basal region of the mandible, and the bone marrow is contained in thick cortical bone. We conducted ex vivo studies using programmed osteotomy and fracture, exploring the transverse and greenstick types of bone fracture, the latter of which allows for slight bending and could result in a slightly convex contour compatible with the mental region. The vestibular to lingual V-ostectomy proved the most acceptable method and was adopted. The V-wedge was then removed, separating the graft into two adjacent sections. The graft was then bent and approximated to a triangular arch form and stabilized with a standard metal wire. As stressed by Kokosis et al. [14], formatting of the fibula graft was a critical step during initial implementation of this surgical method, which has now evolved with technologies such as virtual planning, thus allowing the preparation of three-dimensional models of grafts prior to surgery.

In accordance with the technique described by Taylor et al. [8], a 1-cm sleeve of muscle was kept around the circumference of the fibula in order to preserve blood supply. We consider this combination of periosteum, muscle, and blood vessels as an adequate elastic mechanical structure to restrict the bone segments, while also enabling the physiological process of formation of bone callus after fracture, leading to local healing.

A restricted periosteal detachment allowed access to the cortical surfaces. In planning the osteotomies for this procedure, in addition to the number and location of the segments, we considered the length and positioning of the vascular pedicle near the future mandibular angle. In contrast to the current availability of various tools, no *in situ* preparation existed 38 years prior. The preparation time of this first fibula graft, involving segmentations, fixations, and anastomoses, was approximately 6 h. According to Taylor et al. [8], an ischemia time between 1 h 15 min and 2 h 30 min, with 10 to 12 h duration of the procedure, does not compromise total survival of the graft. A recent review on mandible reconstruction with free fibula flaps showed that a total ischemia time between 1.2 and 5.0 h, along with surgical time between 7.0 and 15.5 h, is not significantly associated with major complications [15].

The fibular pedicle was irrigated with heparin solution before anastomoses, as recommended at the time, while the onlay technique was used for fixation of the graft on the recipient mandible. After correcting for excess bone length, the medial cortical region of the mandibular component and the lateral cortical region of the fibula were both removed, creating low profiles for bone placement. The fibular profile was better established and geometrically delimited. Bone neoformation occurs between the two vital bone surfaces approximated to one another, levelled longitudinally at the external face and superimposed at the medial surface with proximal elongation, offering a larger transversal bone volume. Fibular reinforcement of the recipient jaw was also suggested by Hurczulack et al. [16]. In a proposal to use a male-female joint on the external cortical surface of the mandible, thus preserving the lingual plate, fixation of the graft by miniplates resulted in better stability.

Our previous studies with mandibular osteotomy [17, 18] suggest that an increase in the contact area of the proximal segment, and consequent virtual decrease of the distal arm, results in lower tension and displacement, as well as greater stability with rigid internal fixation. In the present case, only one 2.0 miniplate and 4 (L) plus 6 (R) screws were used in the external face, and intermaxillary immobilization was not performed. This procedure was also reported by Menard et al. [19], who used four osteoseptocutaneous fibular flaps for mandibular reconstruction and stressed the use of rigid fixation with miniplates, without intermaxillary immobilization.

Various types of reconstruction plates and miniplates have been tested for fixation of free fibula flaps. In a retrospective analysis of 143 mandibular reconstruction patients, no relationship was observed between the use of reconstruction plates or miniplates and complication rate [20]. Similarly, a meta-analysis including 511 patients showed no differences in the use of these two systems of fixation; however, clinical records showed that reconstruction plates were associated with both less complications and local exposure in the postoperative period than miniplates [21]. Parise et al. retrospectively evaluated 43 patients submitted to mandibular reconstruction with free fibular flap [22]. In the two cases in which system 2.4 reconstruction plates were used for fixation, the plate was exposed to the oral cavity during the postoperative period. Among the other 41 patients who received miniplates, only four (9.3 %) were exposed. An additional study including 544 patients who received fibular grafts showed that miniplates are advantageous for presenting a lower profile and using screws of smaller diameters, thus decreasing their risk of exposure [23].

Recent advances in surgical planning using computer-aided design to manufacture (CAD/CAM) customized fixation plates represents a valuable improvement in mandibular reconstructive surgery [24].

The fibula can be adapted to correct different types of bone defects. However, the height of the fibula may not be adequate for vertical leveling with the residual receptor bone or for sequential alignment of the segments. In the present case, performance of a preprosthetic surgery with vestibulo-lingual sulcoplasty two years later, associated with a free skin graft according to Obwegeser [25], allowed the comfortable use of a total dental prosthesis. The soft tissues surrounding the fibular bone structure were retracted by incision and supraperiosteal divulsion in the alveolar ridge area and maintained at the level of the lower bone edge. A split thickness skin graft was removed from the anterior region of the thigh. The periosteal and epithelial bloody surfaces were transported and maintained superimposed by an acrylic tray, favoring the process of plasmatic imbibition and inosculation of blood vessels [26, 27] and leading to scarring.

Fourteen years later, the patient searched for an osseointegrated implant, a procedure which had been implemented by that time. Surprisingly, in the relevant clinical evaluation, we found a flat wide edge in the cortical bone surface, which was now very similar to an edentulous, ovoid-type mandible. This structure, however, did not have an adequate height for osseointegrated implant fixation. To improve its thickness, we planned a ¨sandwich¨ graft in the anterior region with iliac crest autogenous bone. The preparation of the receptor area involved longitudinal osteotomy and mobilization of the upper cortical segment, followed by interposition of a left and right cortico-medullary bone block fixed with microplates and screws. The stable result was an approximately three-fold increase in the cortical height that had established during the long permanence of the grafted fibula, allowing the osseointegrated prosthetic rehabilitation that is still fully functional today.

The fibula flap method reported by Taylor et al. in 1975 [8] continues to be applied as originally described, especially where soft tissue damage is not extensive. Our group reported the use of this method for the repair of leg defects in 1981, with a detailed description of the microsurgical technique [28]. Use of this method in reconstructive surgery has been expanded by later advancements, such as the inclusion of a skin island to monitor viability of the flap as described by Yoshimura et al. in 1983 [29], clinical experiences with osteomyocutaneous flaps that have confirmed the endosteal and periostal blood supplies and enabled multiple osteotomies, as reported by Chen and Yan in 1983 [30], and the use of osteoseptocutaneous flaps by Wei et al. in 1986 [31]. Mandibular reconstruction with free osteoseptocutaneous fibula graft, as described by Yim and Wei in 1994 [32], confirmed the vascular anatomy with an extensive double blood supply, i.e., periostal and endothelial, which guarantees the viability of the graft despite the performance of multiple osteotomies.

In 1988, Jones et al. [33] described the “double-barreled” FVFF as a flap folded over its own base, maintaining the original vascularization and consequently having no need for additional anastomoses; this resulted in greater resistance to the structure of the lower limb, which is sometimes very thin. The method was extended, by Horiuchi et al. [34], into the fibula graft for mandibular reconstruction. The mean reported fibula thickness of 1.5 cm is similar to the description of the average 10–12 mm height of the fibula described by Kokosis et al. [14]. This height is considered inadequate to recover the 3–4 cm vertical dimension of the mandible, adding to 1 cm of the dental arch. According to Houriuchi et al. [34], the use of the double-barreled graft provides over 4 cm of alveolar height. Considering these values, our experience with the iliac crest graft in this case was rewarding, allowing the desired prosthetic rehabilitation.

In a sequence of publications, Sassi and collaborators presented promising variations of the double-barreled fibula autograft method used in total or partially edentulous areas, thus providing better conditions for subsequent implant-supported prosthetic rehabilitation in a single procedure [35–37]. Navarro Cuéllar et al. [38] compared various graft techniques, showing that double-barreled and iliac crest grafts present better stability in bone height, lower bone resorption, and higher success rates of the osseointegrated implant compared to vertical distraction. The authors also observed that, in patients undergoing radiotherapy, bone reconstruction was not affected, although the implants were less stable.

Since this first case of FVFF use in 1985 [11], the procedure remains the gold standard for mandibular reconstruction. Several advancements have been introduced, including preoperative planning with the use of computed tomography–based stereolithographic models, three-dimensional virtual surgical software, and the creation of patient-specific cutting guides [39]. As such, current flap survival rates are around 87–100 % [40].

Many patients still experience residual difficulties however, especially relating to speaking and eating, as well as to aesthetic considerations that impact quality of life. The WHO defines quality of life as “an individual’s perception of their position in life in the context of the culture and value systems in which they live and in relation to their goals, expectations, standards and concerns.“ For example, in a report of 213 free flap head and neck reconstructions, the overall flap success rate was 93.4 %, although an unrestricted oral diet and intelligible speech were recovered in a lower percentage of patients, 76 % and 88 %, respectively [41]. In a retrospective review of 20 patients who underwent free fibula flap surgery for maxillofacial reconstruction, Alotaibi et al. observed improved outcomes, with 19 resuming a normal diet, 18 achieving good oral opening, and 17 patients with normal speech, normal occlusion, and an aesthetically good result [42]. In the long-term follow up reported here, the patient is satisfied with the functional outcomes of the procedure and has achieved normal feeding, speech, and head movement, with only a light restriction in mouth opening.

Despite a great number of literature reports on the results of mandibular reconstruction with vascularized bone grafts, there is still a lack of evidence relating to the long-term evaluation of the outcomes [43]. The present work represents the longest-term follow-up of a patient undergoing mandibular reconstruction with FVFF, attesting to the excellency of this surgical approach despite being performed without many of the advancements now available, returning the patient to a high-quality life for at least the past 38 years.

## Data Availability

Not applicable.

## Abbreviations

FVFF: Free vascularized fibula flap
